# The Lysine at Position 177 Is Essential to Limit the Inhibitory Capacities of Sprouty4 Protein in Normal and Cancer-Derived Cells

**DOI:** 10.3390/ijms26157353

**Published:** 2025-07-30

**Authors:** Maximilian Schiwek, Kathrin Ruhdorfer, Christoph Pfurner, Hedwig Sutterlüty

**Affiliations:** Center for Cancer Research, Medical University of Vienna, Borschkegasse 8a, A-1190 Vienna, Austria; maximilian.schiwek@chello.at (M.S.); kathrin.dorfer@gmx.at (K.R.); christoph.pfurner@gmail.com (C.P.)

**Keywords:** Spry4, sprouty, FGFR1, lung cancer, osteosarcoma

## Abstract

The Sprouty (Spry) proteins modulate signalling and regulate processes like cellular migration and proliferation. Here, we investigated a Spry4 alteration substituting a lysine at position 177 to an arginine, based on a mutation found in Kallmann syndrome, a genetically heterogeneous disease connected to reduced fibroblast growth factor receptor1 (FGFR) signalling. Using growth curves to evaluate proliferative and scratch assays to determine migrative capacities of the cells, in normal fibroblasts as well as in osteosarcoma-derived cells, we demonstrate that the modified Spry4^K177R^ version hinders both processes, which the unaltered protein cannot do under the same conditions. The inhibition of these processes was accompanied by lower relative phospho-extracellular-signal-regulated kinases (pERK) levels in response to serum induction, indicating that activation of MAPK was less efficient. In contrast to the situation in these cells of mesenchymal origin, in lung cancer-derived cell lines both variants of Spry4 were able to interfere with proliferation of tested cells, and in the cells with elevated FGFR1 expression the Spry4 proteins with an alteration at codon 177 were even more effective. In summary, these data indicate that the lysine at position 177 restricts the ability of Spry4 to inhibit signal transduction at least in cells with high FGFR1 levels.

## 1. Introduction

The fibroblast growth factor receptors (FGFRs) are transmembrane proteins, which harbour an intracellular tyrosine kinase domain and belong to the class of receptor tyrosine kinases (RTKs). The family consists of four family members which are usually expressed as one of two alternative splicing forms. This creates a wide range of signal receivers, which differ from each other, among other characteristics, in their affinities to the multiple members of the FGF family [1]. Upon ligand binding, the FGFRs function as dimers and initiate different signalling pathways such as the mitogen-activated protein kinase (MAPK), the phospholipase C gamma (PLCγ) or phosphoinositide 3-kinase (PIK3) signal cascades [2]. The FGF/FGFR system is an important regulative system involved in coordination of developmental and differentiation processes, and malfunctions within this system are crucially connected to neoplastic disorders [1].

FGFR1 is a receptor that is expressed throughout the human body and shows aberrations in all kinds of cancers [3]. The FGFR1 gene is frequently amplified in diverse cancers including lung, head and neck and urothelial cancer [4]. Furthermore, the occurrence of activating mutations [5] or a different pattern of the alternative splicing forms [6] can contribute to the malignancy of cancer cells. Additionally, genomic recombination can create FGFR1 fusion proteins, which are considered important oncogenic drivers in tumours like gastrointestinal stromal tumour (GIST), glioblastoma, breast cancer, bladder urothelial carcinoma or non-small cell lung cancer (NSCLC) [7]. Mutations in the FGFR1 gene can also cause hereditary syndromes. If the alteration results in a more active form of the receptor, craniofacial and skeletal abnormalities are manifested as hereditable disease [8].

In case of genomic mutations resulting in restricted signal transduction via the FGFR1, congenital hypogonadotropic hypogonadism is the main phenotype observed. In combination with hyposmia or anosmia, the genetically inherited disease is referred to as Kallmann syndrome. The associated disorders are largely missing [9]. The incidence of FGFR1 mutations in Kallmann syndrome is about 10%, and in the last decades many pathogenic mutations in associated genes including Spry4 have been identified in patients [10].

Spry proteins are known inhibitors of signalling cascades induced by RTKs including the FGFRs. In humans, four homologues of the protein initially discovered in drosophila were identified. Knockout mice of Spry1, Spry2 and Spry4 show characteristic phenotypes often similar to the one observed with FGF overdose [11,12,13,14]. A double knockout of Spry2 and Spry4 has a more severe outcome than both of the single knock-outs suggesting that the Spry proteins have overlapping and unique functions [14]. In the cerebellum a knock-out of Spry1, Spry2 and Spry4 led to severe phenotype which could be rescued by reducing the level of FGFR1, thereby verifying the primary function of Spry proteins as inhibitors of FGF [15].

Due to their central function as inhibitors of MAPK activation, Spry proteins can exert tumour-suppressive roles in cancers evolved in different tissues [16], but in other tumour entities Spry protein members fulfil oncogenic functions as shown for example in colon [17] or rhabdomyosarcoma [18]. In glioblastoma, Spry3 [19] and Spry2 [20] have a tumour-promoting potential, while Spry4 was shown to interfere with malignant features of glioblastoma [19]. A tumour-suppressive function of Spry4 was additionally shown in lung cancer [21] and in breast cancer-derived cells [22].

Recently, mutations in the Spry4 were discovered to be associated with congenital hypogonadotropic hypogonadism [23]. Further reports verified the contribution of Spry4 mutations in oligogenic variants of Kallmann syndrome [24,25]. A frequently with congenital hypogonadotropic hypogonadism-associated alteration [23,26] affecting the Raf-binding domain of Spry4 was shown to be a hyperactive inhibitor of cell proliferation and migration [27].

In this manuscript, we aimed to investigate the effects of the Kallmann syndrome-associated Spry4 mutation c530A>G, which creates a lysine to arginine conversion at position 177 of the Spry4 protein. This mutation was found in two independent studies, and in both cases associated with the Kallmann syndrome [23,24]. Therefore, the altered arginine variant was compared with the lysine variant, wild-type (wt), concerning its influence on signalling and the thereby controlled processes of migration and proliferation in different cell types.

## 2. Results

### 2.1. The Spry4 Variant Associated with Kallmann Syndrome Is Capable to Inhibit Migration and Proliferation of Normal Human Fibroblasts

**Figure 1:**
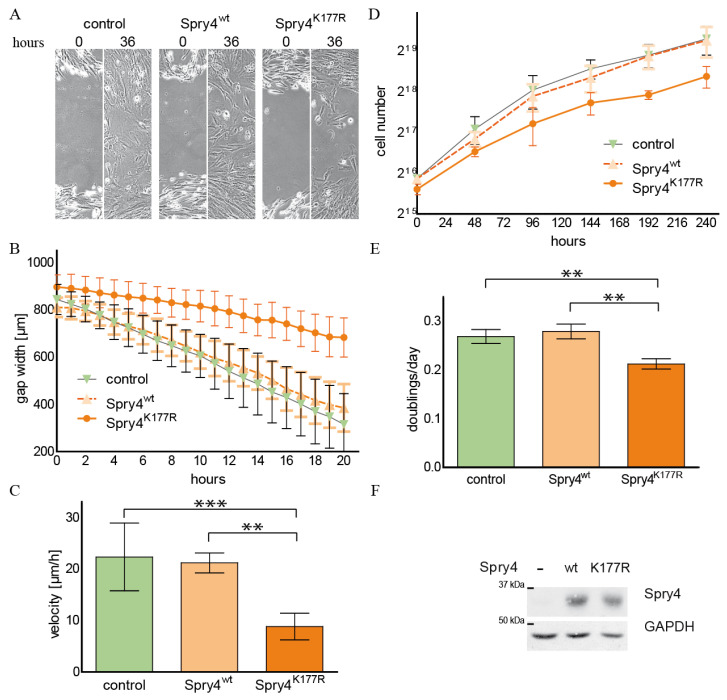
Influence of Spry4^K177R^ on migration and proliferation of normal human lung fibroblasts. WI38 cells were infected with adenoviruses expressing a luciferase and Spry4^wt^ as control proteins or the examined Spry4^K177R^ protein. Then, 24 h later, a scratch assay (**A**–**C**) or a growth curve (**D**,**E**) were performed. For the scratch assay, images were taken every hour. (**A**) Representative images of the gap at the indicated time points are shown. (**B**) The width of the gap was measured every two hours using ImageJ 1.53. A graph combining the measurements of 8 different scratches is depicted in this graph. (**C**) Linear regression of 6 independent experiments (made in duplicates) was performed using the GraphPad Prism 5.0 program. The calculated velocities (mean ± SD) are presented in a bar plot. (**D**) For growth curve analysis, the cell number was determined every second day and data of a representative growth curve are depicted in a graph. (**E**) Using nonlinear regression as growth equation in the GraphPad Prism program, doubling times of 4 independent experiments were calculated. To obtain the doublings per day, 24 h were divided by the doubling time in hours and presented as bar blot. (**F**) Spry4 expression was verified by immunoblot using the indicated antibodies. Glyceraldehyde-3-phosphate dehydrogenase (GAPDH) was used as loading control. Statistical significance was calculated using one-way analysis of variance (ANOVA) with a Tukey post hoc test. ** *p* < 0.001; *** *p* < 0.0001.

During development and in normal regulatory processes within the human organism, Spry4 mainly acts as a negative regulator of growth factor-induced processes [28]. In many studies, this protein is more strongly expressed in the mesenchymal compartment of a tissue [29,30,31]. In our first series of experiments, we therefore wanted to test whether and to what extent a Spry4 protein, which is altered due to a mutation frequently associated with the diagnosis of Kallmann syndrome, influences proliferation and migration of human fibroblasts. For this purpose, normal human lung fibroblasts WI38 were infected with adenoviruses, which contain the coding sequence of either a control protein (luciferase and/or cyan florescent protein CFP), the most commonly expressed, unaltered version of Spry4 (Spry4^wt^) or the Spry4 protein altered by the investigated mutation identified in Kallmann syndrome (Spry4^K177R^). The infected cells were then tested for their migration capacity using a scratch assay. As depicted in Figure 1A, the cells expressing a control protein as well as those producing the Spry4^wt^ protein were able to close the gap carved into the cell lawn by migration after about 36 h. At this time point, the cell lawn of WI38 ectopically expressing the Spry4^K177R^ is still showing a clearly unsealed cut. The presence of Spry4^K177R^ means that the cells concerned are constantly moving slower than those which have picked up a control or a Spry4^wt^ encoding virus (Figure 1B). The migration speed determined by linear regression of the gap width over a time period of 10 h was significantly lower, if Spry4 contained an arginine at position 177 instead of a lysine (Figure 1C).

Like the migration speed, proliferation rate of cells is also linked to signal transduction induced by RTKs, even if Spry proteins do not always have the same influence on the two processes. With the next set of experiments, we aimed to elucidate how the conversion of the lysine at position 177 into arginine modulates cell proliferation. By counting the cells every 2 days, a growth curve analysis of WI38 cells that ectopically express either a control protein, the lysine or the arginine variant of Spry4 was performed (Figure 1D). While WI38 overexpressing the original Spry4^wt^ protein had an unchanged doubling time compared to the control-treated cells, the presence of Spry4^K177R^ retarded the cell division significantly (Figure 1E). As depicted in Figure 1F, adding the respective adenoviruses to the cells resulted in a comparable overexpression of the Spry4 protein variants and was measured 5 days after starting the growth curve.

These data demonstrate that the alteration of the Spry4 gene identified in Kallmann syndrome-diseased people produces a protein variant that is capable of inhibiting RTK-mediated processes in normal human embryonic fibroblasts, a cell type in which the original variant is unable to achieve a detectable effect.

### 2.2. Replacement of the Lysine at the Position 177 by an Arginine Creates a Spry4 Protein Capable of Inhibiting Activation of MAPK Pathway in Normal Human Fibroblasts

**Figure 2:**
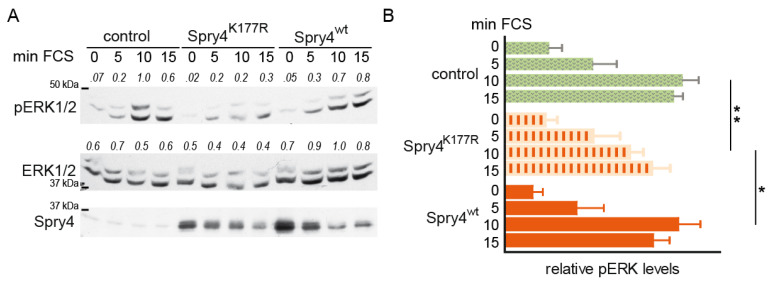
Activation of ERK phosphorylation in the presence of Spry4, its variant harbouring a mutation associated with Kallmann syndrome, and a control protein. Serum-deprived cells were infected with adenoviruses expressing the indicated proteins. Luciferase was used as control. A total of 72 h after serum withdrawal, cells were lysed immediately as well as 5, 10 or 15 min after adding serum. (**A**) A representative immunoblot using specific antibodies against pERK1/2, total ERK1/2 and Spry4 is shown. (**B**) Images were scanned and densitometric analysis was performed using Image Quant. The highest value was arbitrarily set as 1. The blot summarizes the calculated pERK to ERK ratio of 4 experiments (mean ± SEM). Significance was calculated by performing a 1-way ANOVA test in GraphPad Prism. * *p* < 0.05; ** *p* < 0.001.

Spry proteins have been shown to primarily inhibit the activation of the MAPK pathway by growth factors [32]. For this reason, we next investigated how the presence of the two Spry4 variants affects the signal transduction via MAPK cascade by detecting the levels of ERK phosphorylation. In order to address this question, growth-arrested WI38 fibroblasts were infected with the adenoviruses encoding the two Spry4 variants of interest and a control protein. Two days later, serum was added to induce growth factor-dependent signalling. As depicted in Figure 2A, in response to the mitogenic-induced signalling, ERK was phosphorylated and peaked 10 min after the induction. While the fibroblasts expressing Spry4^wt^ showed an ERK activation pattern that hardly differed from those of the control-treated ones, ERK phosphorylation in Spry4^K177R^–expressing WI38 was delayed and/or less pronounced. At the time point 10 min after serum addition, the pERK levels in this cell population were significantly lower than those of the one treated with the two other adenoviruses (Figure 2B), although the extent of expression of Spry4 variants was comparable (Figure 2A).

Summarized, these experiments demonstrated that in normal fibroblasts the investigated mutation found in Kallmann syndrome creates an altered Spry4 protein that is superior to the wt version in terms of its ability to inhibit ERK activation and the connected processes’ proliferation and migration.

### 2.3. The Spry4^K177R^ Variant Is Able to Inhibit Cell Migration and Proliferation in an Osteosarcoma-Derived Cell Line

**Figure 3:**
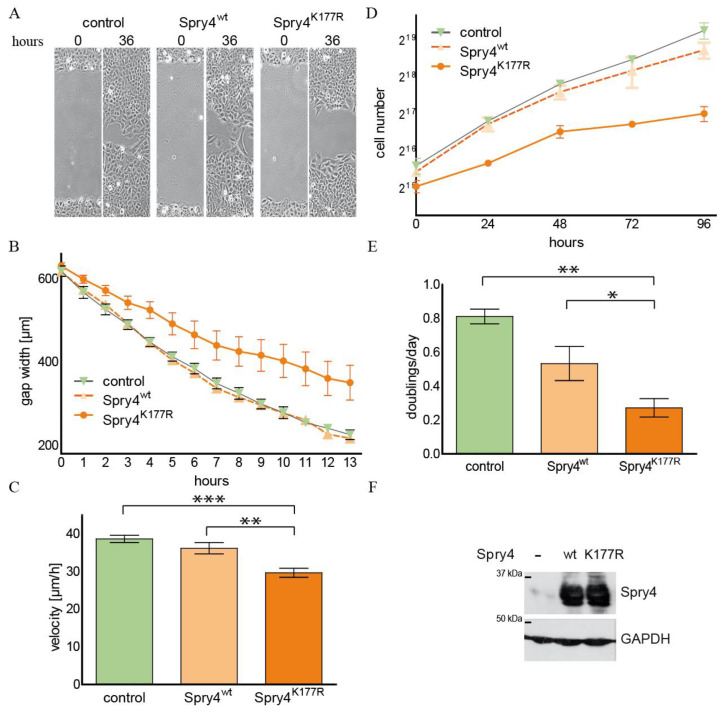
Migration and cell doubling of the osteosarcoma-derived cell line U2OS in the presence of Spry4^K177R^. Cells expressing Spry4^wt^, Spry4^K177R^ or luciferase (control) due to infection with adenoviruses harbouring the respective coding sequences were analyzed 24 h postinfection (pi). (**A**) Brightfield pictures of representative gaps in a U2OS cell lawn using the VISITRON Live Cell Imaging System from Leica taken 0 and 36 h after scratching. (**B**) The measured gap width of an experiment with three scratches was depicted in a line graph and the velocity was determined by linear regression over the first 9 h. (**C**) Display of the calculated velocities. Each bar depicts the mean of 6 independent experiments ± SEM. (**D**) One growth curve performed in triplicates is presented as a graph showing the number of cells as a function of the time elapsed in hours. (**E**) The means of the calculated doublings/day of 5 growth curves are depicted in a bar chart. (**F**). Western blot showing the expression of Spry4 in the analyzed cell populations. GAPDH was used to confirm equal loading. Statistical significance was determined by 1-way ANOVA. * *p* < 0.05; ** *p* < 0.001; *** *p* < 0.0001.

The FGFR1 gene is often amplified in osteosarcoma cell lines and silencing of its expression has been shown to reduce proliferation in osteosarcoma-derived cells [33]. In this cell type, Spry4 expression has failed to interfere with cell proliferation [34]. Therefore, we wanted to examine if a Spry version expected to be hyperactive towards FGFR1-mediated signalling might influence these processes. Using the osteosarcoma-derived cell line U2OS, the effect of the two Spry4 variants on cell migration and cell proliferation was tested and compared to a control-treated cell population. After a scratch was introduced in the cell lawn, U2OS cells closed the arisen gap after 36 h (Figure 3A). If the cells ectopically expressed Spry4^wt^, the time period needed to reclose the lawn was unchanged, while the presence of the Spry4^K177R^ protein inhibited migration of the osteosarcoma-derived cells, so that the gap is still visible at this time point (Figure 3A). Observing the migration over a time period of 13 h revealed that the gap closure is a linear process which is continuously retarded in the presence of mutated Spry4 (Figure 3B). The average migration velocity was significantly lowered from around 35 µm/h to 28.87 ± 3.637 µm/h (Figure 3C).

To evaluate the influence of the Spry4^K177R^ protein on cell proliferation, a growth curve of U2OS cells infected with adenoviruses encoding either one of the Spry4 variants or a control protein was performed. The cells doubled exponentially over 4 days and their cell number accumulated slower if treated with the virus expressing the arginine variant of the Spry4 protein (Figure 3D). The doubling time was increased twofold compared to the control and Spry4^wt^-expressing U2OS cells (Figure 3E). Expression of Spry4 was comparable between the wt and the mutated variant (Figure 3F).

These data support the hypothesis that a Kallmann syndrome-associated mutation causes hyperactivity of the negative FGF-regulator Spry4 and demonstrate that this version is additionally able to interfere with RTK-mediated processes in malignant cells.

### 2.4. Like in Normal Fibroblasts, MAPK Activation Is Weakened in Presence of the Mutated Spry4 Version in Osteosarcoma-Derived Cells

**Figure 4:**
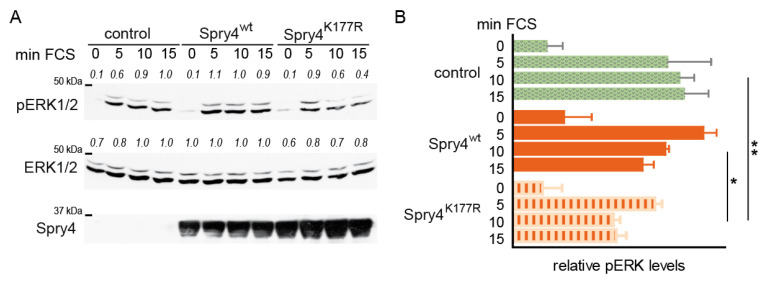
Determination of the pERK/ERK ratio in normal fibroblasts in order to obtain an indicator of the excitability of the MAPK cascade. Serum-deprived U2OS cells expressing Spry4^wt^ and Spry4^K177R^ or luciferase as a control were induced by the addition of FCS 48 h pi. (**A**) Western blot detecting pERK, ERK or Spry4 at the time points 0, 5, 10 and 15 min after induction. (**B**) A densitometric analyses of the intensity of the bands was performed. The highest value of the control group was arbitrarily set as 1. The bar chart summarizes three experiments. Statistical analyses were calculated by comparing each time point between the 3 groups with 1-way ANOVA using a Tukey post hoc test. * *p* < 0.05; ** *p* < 0.001.

Although U2OS cells are malignant, activation of the MAPK pathway is still fully functional if serum is added to starved cells [34]. To evaluate the cellular capacity to phosphorylate ERK, cells were serum starved and infected with adenoviruses expressing a control or a Spry4 variant protein before signalling was induced by the addition of serum. As depicted in Figure 4A, the levels of phosphorylated ERK increased immediately after serum addition and already after 5 min the maximal induction was observable. If Spry4^K177R^ was present, the levels dropped already after 10 min and were significantly lower than in the Spry4^wt^-expressing and control-treated cells (Figure 4B).

These data demonstrate that concomitant with the inhibition of proliferation and migration, Spry4 with a substitution of lysine 177 to arginine interferes with serum-mediated signalling inducing ERK phosphorylation.

### 2.5. In a Lung Cancer-Derived Cell Line with an Activating EGFR Alteration, Both Spry4 Variants Are Equally Strong Inhibitors of Cell Proliferation and Migration

**Figure 5:**
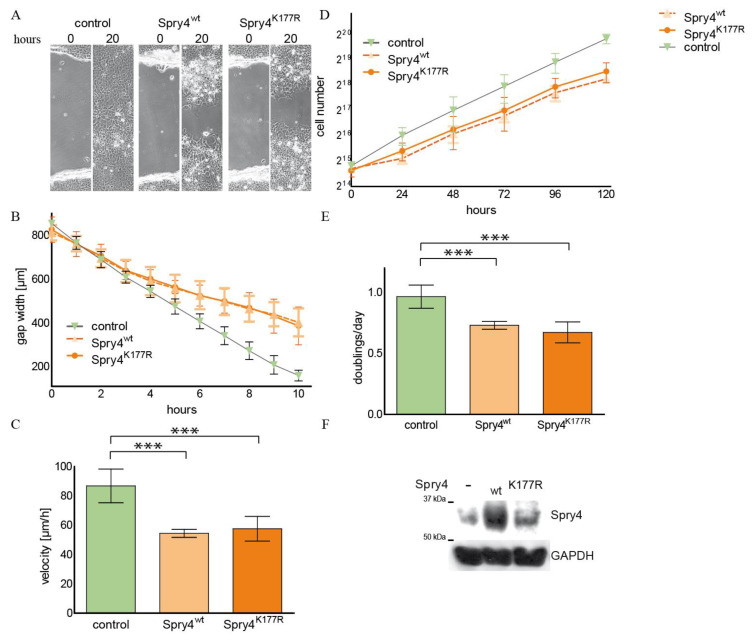
Scratch and growth curve assays of CRL2686 ectopically expressing the Spry4 variants. The lung adenocarcinoma-derived cells were coerced to express either Spry4^wt^ or Spry4^K177R^. As controls, lacZ and/or luciferase proteins were used. (**A**) A series of pictures of the cells expressing the designated proteins before and 20 h after application of a scratch. (**B**) The hourly measured distances between the migration fronts were plotted against the time in a line graph. (**C**) Using linear regression by GraphPad Prism program, the velocity of 6 independent assays (performed in triplicates) were calculated. (**D**) Cells were counted daily over a time span of 120 h and the numbers of 3 to 5 independent experiments are shown in the form of a line diagram. (**E**) Bar chart illustrating the doublings per day of CRL2868 cells expressing a control, Spry4^wt^ or Spry4^K177R^ protein. Each bar represents the mean ± SD of 6 experiments that were performed independently. Statistical significance was established by 1-way ANOVA post hoc Tukey test. *** *p* < 0.0001. (**F**) Using immunoblot, the levels of Spry4 were visualized.

Lung carcinoma is a tumour entity where Spry4 is shown to interfere with the malignant phenotype of the cells [21]. For this reason, it seemed appropriate to carry out an analysis to evaluate if the Spry4^K177R^ is also hyperactive under the circumstances that make up the malignancy of lung cancer cells. For the first series of experiments CRL2868 cells, known to harbour an activating alteration in the EGFR, were chosen [35]. Ectopic expression was achieved by infection with the respective recombinant adenoviruses, and migration of the cells in a scratch assay revealed that, compared to the control cells, the presence of each of the two Spry4 forms inhibited closure of the produced gap within 20 h, a period of time that was sufficient for the control CRL2868 cells to restore the lawn (Figure 5A). The successive closing of the gap in presence of the Spry4 proteins occurred almost synchronously when the distance between the two fronts was observed. In contrast, the control-treated cells were evenly faster (Figure 5B) and their speed, calculated by linear regression of the distance covered per time, was significantly higher at 86.81 ± 11.5 µm/h than of cells expressing one of the Spry4 variants. In both cases, an average velocity of around 55 µm/h was calculated (Figure 5C).

Accordingly, the growth curve shows that the slope of the cell number per hour in the control-treated CRL2868 cells was much steeper compared to the parallel determined increase in the cell population expressing one of the Spry4 protein variants (Figure 5D). Again, there was no obvious difference between the doublings per day as calculated for the two Spry4-expressing study groups (0.73 ± 0.03 and 0.67 ± 0.03 doublings/day for Spry4^wt^ and Spry4^K177R^, respectively), while the control cells doubled almost once (0.97 ± 0.02) per day (Figure 5E). Ectopic expression of Spry4 in CRL2868 cells was achieved in each experiment (Figure 5F).

These data suggest that in lung adenocarcinoma-derived cells the strong inhibitory effect of Spry4 is not further augmented by the substitution of the lysine at position 177 by an arginine.

### 2.6. Spry4^K177R^ Can Exacerbate the Inhibitory Effect of the Wt Variant in a Lung Cell Line Known to Be Dependent on FGFR1-Signalling

**Figure 6:**
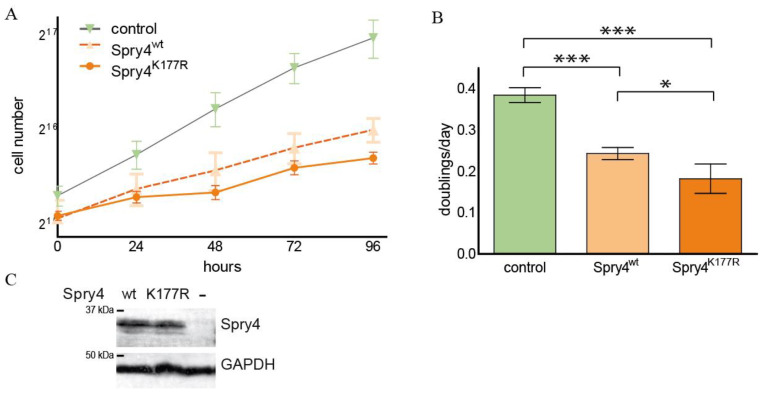
Cell proliferation of Spry4^wt^ and Spry4^K177R^-expressing DMS114 cells. The lung cancer-derived cells DMS114 were incubated for approximately 24 h with adenoviruses expressing the indicated proteins. Luciferase served as control. Subsequently, growth curves were performed. (**A**) Cell numbers were determined for 96 h and a semi-logarithmic diagram with a binary logarithm frame of an individual representative experiment is depicted. (**B**) Three experiments were performed and the collective doubling time was calculated by non-linear regression using the growth curve equation in GraphPad Prism. The results of three independent experiments are presented. One-way ANOVA with Tukey post hoc test was applied for statistical analysis. * *p* < 0.05, *** *p*< 0.0001. (**C**) An immunoblot verifying the ectopic expression of Spry4 using the indicated virus.

In addition to the alterations in the EGFR, that, like the mutations of K-Ras, are predominantly found in adenocarcinoma [36], in other lung cancer entities, FGFR1 amplifications are identified frequently [37]. Therefore, we selected the DMS114 cell line, a small cell carcinoma-derived cell line, known to have an amplification at the genomic locus harbouring *FGFR1* gene [38], to perform growth experiments comparing the two Spry4 variants. As depicted in Figure 6A, like it is reported for lung cancer-derived cells [21], Spry4 expression interfered with cell accumulation upon time if compared with control-treated cells. Nonetheless, the proliferative capacities of DMS114 cells were even further inhibited if the ectopically expressed Spry4 has a substitution of the lysine at position 177. This initial observation was verified by further experiments. On average, cells expressing Spry4^wt^ need about 4 days to reproduce (0.24 ± 0.01 doublings per day), while in the presence of Spry4^K177R^, cells of the same origin divided every five days (0.18 ± 0.02 doublings per day). Control-treated cells multiplied in less than 3 days (see Figure 3B). The expressions of both Spry4 variants were comparable (Figure 6C).

The antiproliferative capacities of the Kallmann-associated Spry4 mutant exceed those of the corresponding wt, when expressed in DMS114, a lung cancer-derived cell line where FGFR1 amplification was identified as a driver mutation.

These data indicate that irrespective of the cellular susceptibility to the inhibitory function of Spry4 protein, the FGFR1-mediated signalling could be a prerequisite for the hyperactive effect of the Spry4^K177R^ variant.

**Figure 7:**
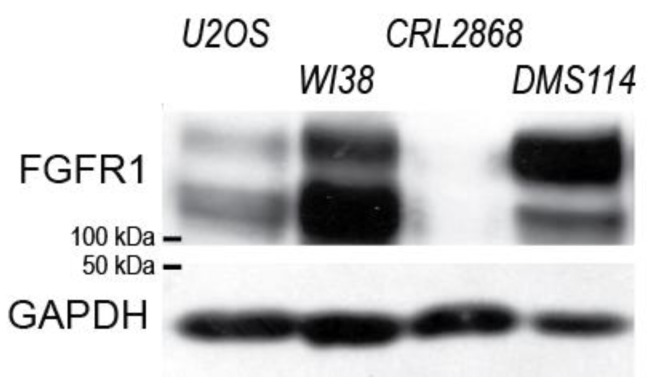
FGFR1 expression in the investigated cell lines. Logarithmically growing cells were lysed and the extracts obtained were analyzed by immunoblotting using the indicated antibodies.

To assess this possibility, we determined FGFR1 expression levels in the investigated cell lines. Thereby, we found that all the investigated cell lines in which the presence of the Spry4 variant identified in Kallmann syndrome inhibited RTK-mediated processes more potently (U2OS, WI38, DMS114) also showed increased protein levels of FGFR1 compared to CRL2868, where no difference in the activity of both variants was observed (Figure 7).

Based on these data, one would assume that rather endogenous level of FGFR1 than tissue origin predisposes a certain cell towards a hypersensitivity to the Spry4^K177R^.

## 3. Discussion

In the study presented here, we have explored the nature of a lysine to arginine substitution on position 177 of the human Spry4 protein. This special derivative is derived from a mutation associated with Kallmann syndrome [39], a morphological disorder often caused primarily by genomic alteration resulting in reduced FGFR1 signalling [10]. Therefore, the assumption is that the created variant of the RTK inhibitor Spry4 generates a hyperactive protein at least against FGFR1-mediated cellular processes, even though the alteration is located in a functionally unexplored region outside of previously defined domains.

Accordingly, we have actually found that the arginine variant of Spry4 is inhibiting cell proliferation and migration in normal human embryonal fibroblasts WI38, cells which are not affected by the presence of the original unaltered protein. A comparable influence on these two cellular key processes could also be observed in the presence of a Spry4^S241Y^ variant which was created as an adaptation of another mutation found in patients suffering from the same syndrome, albeit in that case the genetic alteration is located in the essential well-known RAF binding domain [27]. Consistent with the results obtained in that study, the unaltered version of Spry4 fails to interfere with proliferation and migration in lung fibroblasts [27], while in the same cells Spry2 plays an important role in regulation of these biological processes [39]. In line with this observation, studies in cells from the pancreas [40] show that Spry4 at least in terms of reducing cell proliferation rate is not effective. Similar observations were made in malignant cells derived from epithelial ovarian cancer [41] and osteosarcoma [34].

Although in osteosarcoma-derived cell lines Spry2 protein shows excellent tumour-suppressing properties, Spry4 neither interferes with cell proliferation nor migration in the same experimental setting [34]. Our results confirmed the reported data concerning the inability of Spry4^wt^ protein to interfere with cell proliferation and migration of the osteosarcoma-derived U2OS cells, but additionally, the experiments show that a variation at position 177 (lysine to arginine) enables the Spry4 protein to function as an efficient inhibitor of these processes. This corresponds to the observations made earlier with the Spry4^S241Y^ variant that is associated with Kallmann syndrome [27]. The observation that this cell line expresses high levels of FGFR1 fits with the data reported earlier by others that these cells are particularly vulnerable to treatment with inhibitors targeting this receptor subspecies [42].

Together these observations indicate that the Kallmann-associated missense mutation changing the lysine at position 177 of the Spry4 protein to an arginine creates a version of the protein with an expanded spectrum of cells in which it can function as an inhibitor of known RTK-driven cellular processes. If the presence of the original unaltered version of Spry4 in these mesenchymal-derived cell types has had a barely perceptible effect, it is an effective inhibitor of cell proliferation and migration in breast cancer [22] and glioblastoma-derived cells [19]. Additionally, via a mechanism involving c-Src-mediated expression of beta3-integrins, migration of endothelial cells is decelerated in the presence of Spry4 protein [43]. It has also been shown that proliferation of colon cancer-derived cells slows down in the presence of Spry4 [44]. In both normal and malignant cells, it can be observed that higher levels of Spry4 protein both inhibit and promote the proliferation and migration of the modulated cells. In some cases, even no effect is reported [28]. But it is not clear which internal parameters determine the influence of the protein in one or the other direction.

Within this study, we were also able to confirm earlier findings that show decelerated proliferation and migration of lung cancer-derived cells in the presence of Spry4 [21] with our data, in which a strong inhibitory function of Spry4^wt^ was demonstrated in both investigated lung cancer-derived cell lines. Interestingly, the effect could additionally be observed in the cell line CRL2868 harbouring an E746-A750 deletion in the EGFR gene. This genetic alteration is frequently found in lung cancers and is known to sensitize the cells towards EGFR inhibitors indicating dependency of the carrier cells towards ligands to this receptor [45]. Considering that previous studies show that Spry4 is unable to perform its inhibitory functions if signalling is induced with EGF [27,46,47], it would not necessarily follow that in this background Spry4 is slowing down serum-mediated proliferation and migration of the cells. Nonetheless, both Spry4 variants strongly inhibited both RTK-dependent processes in CRL2868 without any noticeable difference. In contrast, cell proliferation of DMS114 cells was slowed down efficiently by both versions of Spry4, but the protein adaptation created based on the alteration found in Kallmann-syndrome patients inhibited the process to a greater extent. In this cell line, too, levels of FGFR1 were high and corroborating earlier reports show that these cells are particularly sensitive towards inhibitors of FGFR1 [48]. Additional studies will be carried out in the near future to find out whether the presence of high quantities on FGFR1 is actually an absolute necessity for the hyper-inhibitory effect of Spry4^K177R^. To summarize, in all those cells in which the modified Spry4^K177R^ played a hyper-inhibitory role, it turned out that a prominent expression of FGFR1 was detected. Therefore, we assume that FGFR1-mediated signalling actually at least increases the probability that the altered version of Spry4 is superior to the original lysine version in terms of the inhibitory function on proliferation and migration. In the future, further studies elucidating the circumstances of the specific interplay of Spry4^K177R^ and FGFR1 may help to better understand the importance of this specific region in Spry4. Concerning understanding Kallmann syndrome, our studies can only contribute to a deeper understanding of the phenotypes that cause this condition in that they confirm the observations already made and further substantiate the hypothesis that this mutation also contributes to the clinical picture by minimizing FGFR1-mediated signalling.

With regard to the mode of action, our data show that the altered Spry4^K177R^ protein inhibits the phosphorylation of the ERK protein as a final target more efficiently in both, in normal fibroblasts as well as in malignant osteosarcoma-derived cells. Previous studies have also observed that, as with our results, the effect of the tested Spry4 protein on the biological processes examined is accompanied by a reduction in the amplitude of ERK phosphorylation [22,27,34,46,47]. It is not clear why this effect occurs specifically in the presence of FGFR1. The association may of course be purely coincidental, but it is also possible that the altered Spry4 protein has a direct influence on the FGFR1, perhaps similar to the mechanisms reported for Spry2. This family member is shown to regulate endocytosis and stability of the FGFR1 in glioblastoma cells [49]. A recent publication showed that knock-out of the Spry1, 2 and 4 proteins changes the expression profile of FGF ligands and thereby causes an upregulation of certain signalling pathways [50]. Such a change in very specific autocrine activation loops could also explain the observed specificity.

Further assumptions regarding the exact effect of the replacement of lysine by arginine at position 177 on the mode of action of Spry4 are very speculative and result exclusively from the special properties of the individual amino acids. Lysine is known as one of the most frequently modified amino acids in living organisms [51]. Beside acetylation, methylation, succinylation, hydroxylation malonylation, glutaryaltion, propionylation, butyrylation, crotonylation and ADP-ribosylation, also sumoylation and ubiquitination are posttranslational alterations of a protein which are added on lysine residues of a protein [51]. In light of this possibility, it is noteworthy that two recently published studies describe a functional reciprocal relationship between the E3 ubiquitin ligase MDM2 and Spry4. On the one hand, Spry4 expression somehow suppresses the expression of MDM2 [52]. Since degradation of p53 is the best studied task of MDM2, it is conceivable that Spry4 could exert an additional inhibitory role also via this pathway. However, the effect via such a mechanism is rather unlikely, since only two of the three cell lines (WI38 and U2OS), where Spry4^K177R^ is a superior inhibitor, have a wt p53. The inverse relationship is documented as an increased expression of Spry4 if MDM2 is reduced [53]. Since protein half-life of Spry4 is not essentially regulated by proteasomal degradation [54] and the expression levels of both Spry4 variants are comparable, we can almost rule out that the lysine at position 177 is an essential polyubiquitination site in order to regulate Spry4 protein stability. On the basis of the available data, the authors of the publication mentioned above also concluded that MDM2 is not connected to the Spry4 degradation but rather influences its localization [53]. A change in localization may influence endocytosis, but compartmentalized signal repression similar to what is described for Spry2 [55] could also contribute to the hyper-inhibitory function of a Spry4^K177R^ variant specifically towards FGFR1-mediated signalling.

Future projects comparing the immunoprecipitant of the two Spry4 variants will identify differences within the interacting protein panel as well as the modifications. This may clarify if the Spry4^K177R^ is a gain of function mutation or whether the substitution of the lysine abolishes a regulatory mechanism necessary to constrain the inhibitory capacity of the original protein.

Considering the frequency of an FGFR1/FGF overstimulation in many cancers, the hyper-inhibitory effect of this Spry4 version on FGFR1-expressing cells is a valuable insight that can be used for the development of therapeutic concepts, especially to combat cancers with this overexpression.

## 4. Materials and Methods

### 4.1. Cell Lines

All investigated cell lines (WI38, CRL2868, U373 MG, U2OS, MG-63, DMS114) were ordered from the American Type Culture Collection (ATCC, Manassas, VA, USA). Cultivation of the cells was performed in Dulbeccos Modified Eagle Medium (DMEM), containing 10% fetal calf serum (FCS), 100 U/mL penicillin and 100 µg/mL streptomycin, at 37 °C and 7.5% CO_2_, using a humidifying incubator. The cell lines were replaced with freshly thawed aliquots after a maximum of three months. Normal human embryonic fibroblasts WI-38 were only used in passages 23 and 24.

### 4.2. Ectopic Expression of the Proteins Using the Adenoviral System

Adenoviruses coding for Spry4^wt^ [34], luciferase [56] were already available. To generate the adenovirus encoding Spry4^K177R^, site-directed mutagenesis using the sense primer 5′-GCTGGACAGGCACTTCTTGCTGTGC-3′, the antisense primer 5′- GAAGTGCCTGTCCAGCTCGGGTGGG-3′ and pADlox Spry4 as template was performed as described [39]. The mutagenesis was verified by sequencing. Using the so generated plasmid adenovirus was generated as described [57].

The appropriate concentration of virus for each cell line was calculated by measuring the OD_260_ of each virus in comparison to a concentration of CFP-expressing virus which is just efficient to infect 90 to 100% of the cells.

### 4.3. Calculation of Cell Doubling Time by Growth Curve Analysis

Growth curves were performed as described [34]. For WI38 10^5^, for CRL2868, DMS114, and U2OS 4 × 10^4^ were seeded to initiate the growth curve.

### 4.4. Migration Velocity Determined by Scratch Assay

For performing the scratch assay 2×, 6×, 7 × 10^5^ and 5 × 10^5^ cells were used for WI38, CRL2868, and U2OS, respectively. The assay and the calculation were the same as before [19], but the average gap width was calculated hourly.

### 4.5. Cell Signalling Assay

Activation of ERK as a consequence of 20% serum addition was determined following the methodology in [27].

### 4.6. Immunoblotting

According to the protocol carried out in [58] antisera recognizing Spry4 [54], pERK 1/2, (CST#9101, Cell Signalling Technology, Danvers, MA, USA), ERK 1/2 (sc-514302, Santa Biotechnology, Dallas, TX, USA) GAPDH (sc-365062, Santa Cruz Biotechnology, Dallas, TX, USA), and FGFR1 (D8E4, Cell Signalling Technology, Davers, USA) were used as primary antibodies. The horseradish peroxidase-coupled swine anti-rabbit IgG (# P0217) or rabbit anti-mouse IgG/HRP (# P0260) were purchased from Agilent Technologies Inc. (Santa Clara, CA, USA).

## Data Availability

All data are included in the article.

## Abbreviations

The following abbreviations are used in this manuscript:

Spry	Sprouty
FGFR	fibroblast growth factor receptor
pERK	phospho-extracellular-signal-regulated kinases
RTK	receptor tyrosine kinases
MAPK	mitogen-activated protein kinase
PLCγ	Phospholipase C gamma
PIK3	Phosphoinositide 3-kinase
CFP	Cyan florescence protein
RAF	rapidly accelerated fibrosarcoma
MDM2	Murine double minute 2
FCS	fetal calf serum
ANOVA	analysis of variance
wt	wild-type
SD	standard deviation
SEM	standard error of the mean
DMEM	Dulbeccos Modified Eagle Medium
ATCC	American-type cell culture
